# Sanitation

**Published:** 1890-10

**Authors:** James W. Putnam


### Progress in Medical Science.

### SANITATION.

By JAMES W. PUTNAM, M. D.

### FOOD SUPPLY.

The direct relation of public health to the food supply is one which makes the purity of canned goods a very important one. In the report of William K. Newton, M. D., Dairy Commissioner, of New Jersey, for 1889, we find startling and important facts, which are abstracted in the *Sanitarian* for July. The ground covered was very complete, and in the point of canned foods, thirteen American samples were examined, all of which were found to be in good condition and free from metals or other dangerous ingredients. Of

107 samples of imported canned foods examined, eighty-eight were found to be adulterated or not standard ; the chief adulterant was copper, which was added to give a green color to the vegetables.

Fifty samples of ground coffee showed only ten were pure.

Ground spices, 649 samples were examined, of which 306 were adulterated or not standard.

Honey, 111 samples were examined ; eighty-three adulterated.

Molasses, sixty-four samples showed thirty-eight adulterated. Chief adulterant glucose ; some samples had been bleached by the use of sulphuric acid and some contained tin.

Pickles, ten samples, three were pure, seven contained copper.

Fruits, jellies, jams and preserves, 192 samples were examined, of which thirty-three were pure and 159 adulterated.

The analysis shows that many of the samples were devoid of any of the fruit mentioned on the label.

As an illustration, a sample of "currant jelly" contained starch, water, acetic acid, currant flavoring extract, glucose and coloring matter ; the currant flavoring matter is made from amylic ether.

Of this fraudulent jelly stuff, the *Sanitarian* says : "One Philadelphia firm sold thirty-nine tons in one week ; one other factory delivered 1,000 tons in six months. There are about fifty of these factories in the United States, so that one can see the enormous amount of this fraudulent stuff which is eaten by the poorer class of people.

The following table will show the articles used in the fraudulent jams and jellies. Articles used to give substance: Apple pomace, apple juice, starch, glue, gelatine, Japanese isinglass. Articles used as flavors : Apple inferior or spoiled fruit juices, artificial flavors, and the compound ethers, acetic acid.

Artificial colors used : The aniline dyes — eosine, fuchsine, Bismarck brown, garnet red, ruby red and various carmines.

Among the firms whose names were on the labels were two which we recognize of especial interest to physicians in this State, viz.: The Erie Preserving Company and Empire State Preserving Company.

Bologna sausage : Twelve samples were examined, with the result as follows :

The analysis of the bologna and the skin in which the meat was placed showed that some dye, probably one of the anilines, was used to color the material, in order that some defect might be hidden or the article made to appear better than it really was ; also, that some substance had been applied to the exterior of the sausage similar to varnish. Further analysis revealed the presence of triamidoazobenzine, or Bismarck brown, one of the aniline colors; this was in the meat. The skin, or "casing," was coated with a varnish containing shellac. This discovery was the means of arriving at all the details of the process employed. The sausage in question was prepared in the following way: After the meat was chopped and the sausage-meat thus prepared put into the casings, the sausage was boiled in a bath containing a portion of the following coloring agent: Bismarck brown, fourteen parts; garnet red, two parts; water, one and one-half pints — this gave the sausage a brown color. When this process was complete, the sausages were coated with a varnish composed of shellac, resin, oil, and alcohol.

In order that the small local manufacturers of sausage might engage in the practice of making dyed sausages, the compositions referred to above were offered for sale through the State, and the staining material was sold under the name of "smokine" or "liquid smoke."

The sale of the article was checked by the official action of the inspectors throughout the State.

---

The Danger Limit to Infectious Diseases.—The *American Practitioner*, for July 19th, publishes the following table drawn up by the Society of Public Hygiene, in Paris, for the guidance of the masters of public schools with reference to the time that may be allowed to intervene between the onset of the malady in a pupil, and the date that he may be re-admitted into the school:

| MALADY. | PERIOD OF INCUBATION. | PERIOD OF INVASION. | PERIOD OF RE-ADMISSION THAT MAY BE AUTHORIZED. |
|---|---|---|---|
| Scarlet-fever | 7 | 2 | Forty days from the first day of invasion. |
| Measles | 9 | 4 | Twenty-five days from the first day of invasion. |
| Whooping Cough | 12 | 8 | Thirty days after disappearance of the characteristic cough. |
| Diphtheria | 5 | 2 | Forty days from the first day of invasion. |
| Mumps | 18 | 2 | Twenty-five days from the first day of invasion. |
| Varicella | 14 | 2 | Twenty-five days from the first day of invasion. |

---

There would appear to be a recurrence of the influenza in Vienna. Several cases are stated to have occurred in the hospitals there. In Paris, also, the disease is said to be reappearing.—*Journal of the American Medical Association.*

